# P-1582. Comparing Contamination Rates in Single-Site vs Multi-Site Blood Culture Sampling

**DOI:** 10.1093/ofid/ofae631.1749

**Published:** 2025-01-29

**Authors:** Abhilasha Borad, Ahmed Abdul Azim, Susan Boruchoff, Ali Ibrahim, Melvin P Weinstein, Keith S Kaye

**Affiliations:** Beth Israel Deaconess Medical Center, Boston, Massachusetts; Rutgers Robert Wood Johnson Medical School, New Brunswick, New Jersey; Robert Wood Johnson University Hospital, New Brunswick, New Jersey; Rutgers University, Edison, New Jersey; Rutgers Robert Wood Johnson Medical School, New Brunswick, New Jersey; Rutgers Robert Wood Johnson Medical School, New Brunswick, New Jersey

## Abstract

**Background:**

The utility of blood cultures relies on optimal sampling - two samples obtained from separate sites at different times. We noted repeated instances where, according to the electronic medical record (EMR), 2 sets of blood cultures were obtained in the emergency department (ED) with identical time stamps. When these cultures, presumed to be from a single site, return positive for commensal organisms in both sets, treatment might be initiated for a probable contaminant. This study aimed to compare the recovery rate of commensal organisms in both sets of blood cultures obtained from presumed single-site sampling (SSS) versus multi-site sampling (MSS).

**Figure:**
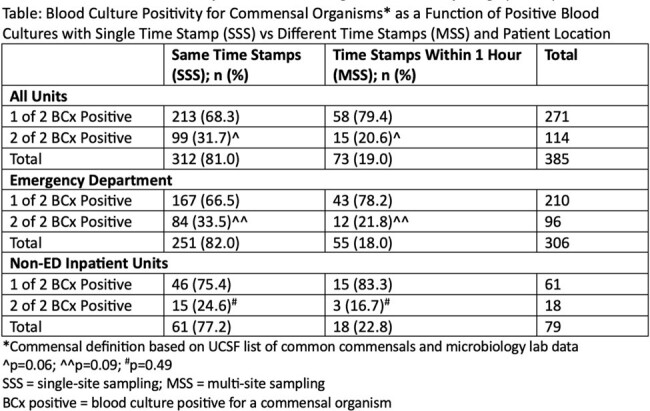

**Methods:**

This is a retrospective analysis utilizing data from microbiology and hospital administrative databases. The incidence rates of blood culture contaminants in patients who had two sets of blood cultures drawn with an identical time stamp for both sets (aka SSS) and in patients who had different time stamps within 1 hour of each other for each set (aka MSS) were compared. Rates from ED and selected non-ED inpatient locations were also compared.

**Results:**

There were 1500 positive blood cultures from 720 patients, and 385 patients had 2 sets of blood cultures drawn with at least 1 positive culture for a commensal pathogen (Table). 312/385 patients (81.0%) had samples obtained via SSS and 73 (19.0%) via MSS. Of the 312 SSS patients, 99 (31.7%) had 2/2 sets positive for a commensal, while 15 of 73 MSS patients (20.5%) had 2/2 sets positive for a commensal (p=0.06). In the ED, 251 patients had samples obtained via SSS and 55 via MSS. 84/251 SSS patients (33.4%) had 2/2 sets positive for a commensal, while 12/55 MSS patients (21.8%) had 2/2 sets positive for a commensal (p=0.09).

**Conclusion:**

Blood cultures obtained via SSS had higher rates of 2/2 set positivity for a commensal as compared to cultures obtained via MSS; however, this study was underpowered to demonstrate statistical significance. Other limitations include the assumption that EMR time stamp for collection of blood cultures accurately represents blood culture collection time. Diagnostic stewardship opportunities include potential EMR modifications and/or additional training related to documentation of timing of blood culture collection.

**Disclosures:**

**Melvin P. Weinstein, MD**, Helixbind: Advisor/Consultant|Safeguard Biosystems: Advisor/Consultant|Selux Diagnostics: Advisor/Consultant **Keith S. Kaye, MD, MPH**, Allecra: Advisor/Consultant|CARB-X: Advisor/Consultant|GSK: Advisor/Consultant|Merck: Advisor/Consultant|Shionogi: Advisor/Consultant|Spero: Advisor/Consultant

